# Single-step assembly of polymer-lipid hybrid nanoparticles for mitomycin C delivery

**DOI:** 10.1186/1556-276X-9-560

**Published:** 2014-10-08

**Authors:** Yunfeng Yi, Yang Li, Hongjie Wu, Mengmeng Jia, Xiangrui Yang, Heng Wei, Jinyan Lin, Shichao Wu, Yu Huang, Zhenqing Hou, Liya Xie

**Affiliations:** 1 The Affiliated Southeast Hospital of Xiamen University, Xiamen University, Zhangzhou 363000, China; 2 Department of Biomaterials, College of Materials, Xiamen University, Xiamen 361005, China; 3 Department of Chemistry, College of Chemistry and Chemical Engineering, Xiamen University, Xiamen 361005, China; 4 Department of Pharmacy, School of Pharmaceutical Science, Xiamen University, Xiamen 361005, China; 5 The First Affiliated Hospital of Xiamen University, Xiamen 361003, China

**Keywords:** Cancer chemoprevention, Controlled release, Drug delivery systems, Phospholipids, Self-assembly

## Abstract

Mitomycin C is one of the most effective chemotherapeutic agents for a wide spectrum of cancers, but its clinical use is still hindered by the mitomycin C (MMC) delivery systems. In this study, the MMC-loaded polymer-lipid hybrid nanoparticles (NPs) were prepared by a single-step assembly (ACS Nano 2012, 6:4955 to 4965) of MMC-soybean phosphatidyhlcholine (SPC) complex (Mol. Pharmaceutics 2013, 10:90 to 101) and biodegradable polylactic acid (PLA) polymers for intravenous MMC delivery. The advantage of the MMC-SPC complex on the polymer-lipid hybrid NPs was that MMC-SPC was used as a structural element to offer the integrity of the hybrid NPs, served as a drug preparation to increase the effectiveness and safety and control the release of MMC, and acted as an emulsifier to facilitate and stabilize the formation. Compared to the PLA NPs/MMC, the PLA NPs/MMC-SPC showed a significant accumulation of MMC in the nuclei as the action site of MMC. The PLA NPs/MMC-SPC also exhibited a significantly higher anticancer effect compared to the PLA NPs/MMC or free MMC injection *in vitro* and *in vivo*. These results suggested that the MMC-loaded polymer-lipid hybrid NPs might be useful and efficient drug delivery systems for widening the therapeutic window of MMC and bringing the clinical use of MMC one step closer to reality.

## Background

Nanotechnology represents a powerful tool in the field of medicine to deal with cancer [1,2], which is still a leading cause of death worldwide [3]. Nevertheless, there is the limited availability of nanotechnology-based drug formulation which is approved by FDA in clinical use. It is a formidable challenge to develop the smarter and better nanoscaled drug delivery systems. Up to now, both lipid-based and polymer-based nanoscaled drug delivery systems have represented two dominant classes of nanoscaled drug delivery systems for anticancer drug delivery and shown promising efficacy for clinical treatment [4-6]. Liposome, one of the most investigated lipid-based carriers, is a spherical vesicle composed of lipid material and has been widely used for drug delivery due to improved safety profile, high delivery efficiency, favorable pharmacokinetics, and ease of surface modification [7]. However, their clinical uses by intravenous injection are significantly limited because of the insufficient drug loading, burst drug release, instability, and toxicity. Polymeric nanoparticles (NPs), one of the most investigated polymer-based carriers, have small particle size, ability to load drug of poor solubility and permeability, appropriate physiological stability, well-storage stability, controlled and sustained drug release, and long systemic circulation time [5,8]. In addition, the preparation of polymeric NPs by nanoprecipitation or emulsion is simple and scalable [9]. However, the biocompatibility of the polymeric NPs formed by most synthetic polymers is not as high as the liposomes, especially at the cellular and animal level. Thus, it is necessary to develop a novel and practical drug carrier which can combine the desired advantages and avoid the undesired disadvantages of both the lipid-based and polymer-based carriers for clinical application [10].

In recent years, the polymer-lipid hybrid NPs designed to merge the best of both worlds had been reported in several groups. The first was that the polymer-lipid hybrid NPs were prepared via the fusion of the polymeric NPs and liposomes [11]. The preparation of the polymer-lipid core-shell structure usually required a two-step process: the initial formation of polymeric NPs and subsequent encapsulation of the liposomes [8,12]. However, the process resulted in the technical complexity and the poor control over the final characteristics of the nanoscaled drug delivery systems [8,13]. The second was that the polymer-lipid hybrid NPs were prepared by a single step which combined the nanoprecipitation (or emulsion) and self-assembly method [14,15]. The strategy satisfied the need for the development of precise and predictable hybrid nanoscaled drug delivery systems, which would be convenient for future scaling-up.

Mitomycin C (MMC) is a water-soluble anticancer and antibiotic agent by inhibiting DNA synthesis and nuclear division and has been extensively used to treat various cancer including stomach, liver, breast, pancreas, colon, and bladder cancers [16]. MMC was a very poor substrate for P-glycoprotein and retained activity against many types of P-glycoprotein-mediated multidrug resistant tumor cells [17,18]. However, a major concern of MMC was in the narrow therapeutic index and serious side effects such as severe myelosuppression and gastrointestinal complications [19,20]. Recently, the drug-phospholipid complex had received significant attention [21,22] because of the significant improvement of drug efficacy and safety [23-30]. To address the issue, we prepared the MMC-soybean phosphatidyhlcholine complex (MMC-SPC complex) to increase the effectiveness of MMC [31]. On the one hand, MMC interacted with soybean phosphatidylcholine (SPC) via the non-covalent bonds and resulted in almost 100% complexation efficiency. On the other hand, the amphiphilic character of the drug-phospholipid complex could promote the passive transport from the water environment to the lipid-rich cell membrane to increase the drug permeability and enhance the drug bioavailability and improve the drug effectiveness [32].

**Figure 1:**
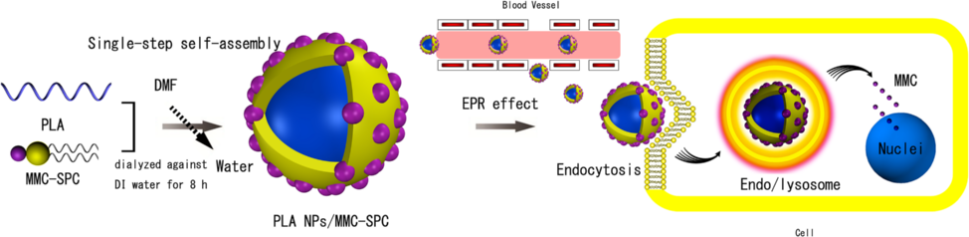
Schematic illustration of the hybrid PLA NPs/MMC-SPC prepared through a single-step method and their drug delivery.

Previously Wang et al. prepared the polymer-lipid hybrid NPs for systemic delivery of small interfering RNA (siRNA) [13]. In this study, to develop the hybrid nanoscaled drug delivery systems that complemented and integrated the characteristics of polymeric NPs and liposomes for systemic MMC delivery, we continued to use the MMC-SPC complex [31] to prepare the MMC-loaded polymer-lipid hybrid NPs by a single-step assembly of polylactic acid (PLA) and MMC-SPC complex (Figure 1). PLA, one of the FDA approved biodegradable polymers for clinical applications, had been demonstrated to be safe *in vivo* for several decades. SPC had always been regarded as a promising candidate for drug preparations due to the biocompatibility, biodegradability, metabolic activity, and low toxicity and cytotoxicity compared to synthetic alternatives [31,33,34]. Most significantly of all, some advantages of the drug-phospholipid complex on the basis of the PLA NPs were as follows. Firstly, the MMC-SPC complex was used as a structural element, which could integrate the best of both polymer and lipid worlds and offer the integrity of the hybrid polymer-lipid nanoscaled drug delivery system. Secondly, the MMC-SPC complex was served as a drug preparation to link the conventional and novel drug formulation, which could increase the lipophilicity, storage stability, efficacy and safety, and control the release of MMC for systemic drug delivery. Lastly, the MMC-SPC complex was acted as a stabilizer/emulsifier to facilitate and stabilize the formation of the nanoscaled drug delivery systems. The advantages of the SPC-emulsified nanoscaled drug delivery systems over those emulsified by the traditional chemical emulsifier poly(vinyl alcohol) (PVA) were investigated. The physicochemical characterizations of the PLA NPs/MMC-SPC were performed by *in vitro* drug release, and *in vitro* and *in vivo* effectiveness was tested in H_22_ cells and H_22_ tumor-bearing mice.

## Methods

### Materials

All chemical reagents were of analytical grade and used without further purification unless otherwise stated. Mitomycin C (MMC; purity grade = 99.5%) was purchased from Hisun Pharmaceutical Co., Ltd. (Zhengjiang, China). SPC was provided by Lipoid GmbH (Ludwigshafen, Germany). Poly(D,L-lactide) (PLA; 10 kDa) was provided by Daigang BIO Engineer Co., Ltd. (Shandong, China). *N*,*N*-dimethylformamide (DMF), 3-(2-benzothiazolyl)-*N*,*N*-diethylumbelliferylamine (coumarin-6), fluorescein 5-isothiocyanate (FITC), and Dulbecco's modified Eagle's medium (DMEM) were purchased from Sigma-Aldrich (St. Louis, MO, USA). Iodide [1,1′-dioctadecyl-3,3,3′,3′-tetramethylindotricarbocyanine iodide (DiR) was provided by Molecular Probes Inc. (Eugene, OR, USA). A dialysis bag (Mw = 8, 000 to 14, 000 Da) was ordered from Greenbird Inc. (Shanghai, China). Deionized (DI) water was used throughout. Fetal bovine serum (FBS) was purchased from Gibco Life Technologies (AG, Zug, Switzerland). Trypsin-EDTA (0.25%) and penicillin-streptomycin solution were from Invitrogen (Life Technologies, Basel, Switzerland). All solvents used in this study were high-performance liquid chromatography (HPLC) grade. Mouse hepatoma cell line H_22_ cells were provided by the Cell Bank at the China Academy of Science.

### Preparation of PLA NPs/MMC-SPC

Firstly, MMC and SPC were added to tetrahydrofuran by vigorous agitation in a 40°C water bath for 4 h [31]. The organic solvent was evaporated under reduced pressure. The MMC-SPC loaded PLA NPs (PLA NPs/MMC-SPC) were prepared by a facile dialysis method. Twenty milligrams of PLA and the weighed amount of MMC-SPC (the weight ratio of MMC and SPC was 1:2) were dissolved in 10 mL of DMF accompanied by vigorous stirring, and then, the resulting organic phase was introduced into a dialysis bag. Subsequently, the dialysis bag was placed into 1,000 mL of DI water as the aqueous phase under moderate agitation. The organic phase was dialyzed against the aqueous phase for 8 h [35], after that, the suspensions were filtered through a 450 and 200 nm polycarbonate membrane (Millipore, Bedford, MA, USA), respectively. Then, the PLA NPs/MMC-SPC were collected by centrifugation with a JA-20 rotor (Beckman Coulter, Inc., Fullerton, CA, USA) at 15,000 rpm at 4°C for 10 min, after that, the PLA NPs/MMC-SPC were resuspended in DI water. The PLA NPs/MMC were prepared by a double emulsion solvent-evaporation technique for comparison. PLA was added into 10 mL of DMF to form the oil phase, and MMC was added into 5 mL of DI water to form the inner water phase. The inner water phase was mixed with the oil phase under stirring and then, the mixture was sonicated in an ice water bath to form a primary emulsion. The primary emulsion was added into DI water with 2% (w/v) PVA and homogenized at of 1,000 rpm.

In addition, the micron-sized PLA NPs/MMC-SPC (used for confocal laser scanning microscopy image) could be formulated as follow: FITC conjugated MMC (FITC-MMC; FITC-MMC was synthesized based on the reaction between MMC and FTIC via a thiourea linkage) was prepared for use as a fluorescence probe. And then, FITC-MMC-SPC complex was prepared in a same way as described above. Thirty-six milligrams of FITC-MMC-SPC complex and 200 mg of PLA were dissolved in 10 mL of DMF, accompanied by vigorous stirring. The mixture was dialyzed against 1,000 mL of DI water for 8 h. Then, the micron-sized PLA NPs/FITC-MMC-SPC were collected by centrifugation at 15,000 rpm at 4°C for 10 min, after that, the micron-sized PLA NPs/FITC-MMC-SPC were resuspended in DI water.

### Drug encapsulation efficiency and drug loading content

For determination of drug encapsulation efficiency and drug loading content, the lyophilized PLA NPs/MMC-SPC were dissolved in DMF. The organic solution was filtered and submitted to HPLC analysis. The amount of MMC was assayed using a HPLC (Waters Corporation, Milford, MA, USA) system consisting of a Waters 2695 Separation Module (Waters Corporation, Milford, MA, USA) and a Waters 2996 Photodiode Array Detector (Waters Corporation, Milford, MA, USA) with the following conditions: chromatographic column, symmetry C_18_ column (250 × 4.6 mm, 5 μm); temperature, 25°C; and elution flow rate, 1.0 mL/min. The mobile phase for the determination of MMC was water-methanol (65/35, v/v). The detection wavelength for the determination of MMC was 362 nm. The drug encapsulation efficiency and drug loading content were calculated according to the formula

$$\begin{array}{l} \mathrm{Drug}\;\mathrm{encapsulation}\;\mathrm{efficiency}\;\left(\%\right)\\ \;=\;(\mathrm{the}\;\mathrm{weight}\;\mathrm{of}\;\mathrm{the}\;\mathrm{loaded}\;\mathrm{drug}\\ \;/\mathrm{the}\;\mathrm{weight}\;\mathrm{of}\;\mathrm{the}\;\mathrm{input}\;\mathrm{drug})\;\times \;100\% \end{array}$$

$$\begin{array}{l} \mathrm{Drug}\;\mathrm{loading}\;\mathrm{content}\;\left(\%\right)\\ \;=\;(\mathrm{the}\;\mathrm{weight}\;\mathrm{of}\;\mathrm{the}\;\mathrm{loaded}\;\mathrm{drug}\\ \;/\mathrm{the}\;\mathrm{weight}\;\mathrm{of}\;\mathrm{the}\;NPs)\;\times \;100\% \end{array}$$

### Characterization of PLA NPs/MMC-SPC

The average particle size, polydispersity index (PDI), and zeta potential of the suspension of the PLA NPs/MMC-SPC were performed by dynamic light scattering (DLS) using a Malvern Zetasizer Nano-ZS (Malvern Instruments, Worcestershire, UK). Particle size was evaluated by intensity distribution. The morphology of the PLA NPs/MMC-SPC was visualized by SEM (LEO 1530VP, Oberkochen, Germany) operating at 20 kV and TEM (JEM 1400, JEOL, Tokyo, Japan) operating at 200 kV.

### *In vitro* stability tests

The lyophilized PLA NPs/MMC-SPC were suspended in phosphate buffer solution (PBS) (pH 7.4) and incubated at 37°C for 48 h. The particle size was assayed at 12 h intervals by DLS.

### *In vitro* drug release

The release of MMC from the PLA NPs/MMC-SPC was determined by a dialysis technique. The lyophilized NPs were dispersed in 3 mL of PBS (1/15 M, pH 7.4) buffer solution. The dispersions were transferred to a dialysis bag (Mw = 8,000 to 14,000 Da) and then subjected to dialysis against 10 mL of PBS at 37°C. At a predesigned time interval, all of the PBS buffer solution was withdrawn and subsequently replaced with the 10 mL of fresh PBS after each sampling. The PLA NPs/MMC and free MMC were used for comparison. The release of MMC was determined by a HPLC method as described above.

### Cell culture

H_22_ cells (mouse hepatoma cell line) were cultured in DMEM supplemented with 10% FBS and 1% penicillin-streptomycin. The cells were cultivated in a humidified atmosphere containing 5% CO_2_ at 37°C.

### *In vitro* cellular uptake

To facilitate the observation of cellular uptake, coumarin-6 was used as a hydrophobic fluorescence probe [36] to load within the PLA NPs/MMC-SPC and PLA NPs/MMC, respectively (designed as the coumarin-6-PLA NPs/MMC-SPC and coumarin-6-PLA NPs/MMC, respectively). H_22_ cells were seeded at a density of 1 × 10^5^ cells per well in 6-well plates with their specific cell culture medium. The cells were incubated at 37°C and 5% CO_2_ for 24 h. One-hundred microliters of the coumarin-6-PLA NPs/MMC-SPC and coumarin-6-PLA NPs/MMC at the equivalent coumarin-6 concentration was added and incubated further for 6 h. The cells were washed with PBS, fixed with 4% paraformaldehyde, and stained with Hoechst 33258 (Sigma-Aldrich, St. Louis, MO, USA). The cells were observed using a Leica TCS SP5 confocal laser scanning microscopy (Leica Microsystems, Mannheim, Germany).

### Flow cytometry

To further quantitatively investigate the cellular uptake, coumarin-6 was used as a fluorescence probe to load within the PLA NPs/MMC-SPC and PLA NPs/MMC, respectively (designed as the coumarin-6-PLA NPs/MMC-SPC and coumarin-6-PLA NPs/MMC, respectively). H_22_ cells were seeded in 6-well plates with a density of 2 × 10^5^ cells/mL and incubated for 24 h, and then, the original medium was replaced with the coumarin-6-PLA NPs/MMC-SPC and coumarin-6-PLA NPs/MMC-SPC at the equivalent coumarin-6 concentration. The cells were incubated for the predesigned time at 37°C and then washed with cold PBS and harvested by 0.25% trypsin-EDTA. The harvested cells were suspended in PBS and centrifuged at 1,000 rpm for 5 min at 4°C. After washing and centrifugation, the cells were resuspended in PBS and performed by a Beckman Coulter EPICS XL flow cytometer (Beckman Coulter, Fullerton, CA, USA).

### Intracellular drug delivery

To understand the intracellular distribution of the drug inside H_22_ cells, FITC conjugated MMC (FITC-MMC; FITC-MMC was synthesized based on the reaction between MMC and FTIC via a thiourea linkage [37]) was prepared for use as a fluorescence probe. H_22_ cells were incubated for 24 h and then cultured with the PLA NPs/FITC-MMC-SPC or PLA NPs/FITC-MMC for 12 h at 37°C. The cells were imaged using a confocal laser scanning microscopy. Rhodamine phalloidin was used to stain cytoskeleton. Hoechst 33258 (Sigma-Aldrich, St. Louis, MO, USA) was used to stain nuclei.

### *In vitro* cell viability

The cytotoxicity of the drug delivery systems was measured using a MTT assay (Sigma-Aldrich, St. Louis, MO, USA) according to the manufacturer's suggested procedures. H_22_ cells were exposed to the PLA NPs/MMC-SPC, PLA NPs/MMC or free MMC with different MMC concentrations for 24 h. The data were expressed as the percentage of surviving cells.

### *In vivo* blood circulation

All the animal procedures complied with the guidelines of the Xiamen University Institutional Animal Care and Use Committee. The experiments were performed on adult male SD rat weighing 200 ± 20 g (mean ± SD) from Shanghai Laboratory Animal Center. DiR was used as a near-infrared fluorescence probe to load within the PLA NPs/MMC-SPC and PLA NPs/MMC, respectively [38]. One-hundred microliters of the DiR-PLA NPs/MMC-SPC and DiR-PLA NPs/MMC at the equivalent DiR concentration was respectively injected intravenously through the tail vein of rats. At timed intervals, 200 μL of blood was collected and stored in heparin containing eppendorf tubes at 4°C for further analysis. The plasma was separated from the blood by centrifuging at 2,000 rpm for 5 min. The plasma was diluted with methanol and centrifuged to remove the insoluble solid. At the excitation wavelength of 748 nm, the fluorescence intensity at 780 nm was measured using a microplate reader and the corresponding DiR concentration was calculated according to an established standard curve.

### *In vivo* fluorescence imaging

For *in vivo* imaging, 0.2 mL of the DiR-PLA NPs/MMC-SPC was injected into the nude mice bearing H_22_ tumor via the lateral tail vein. Imaging was performed at 1, 3, and 12 h after injection using a Maestro™ *in vivo* imaging system (Cambridge Research & Instrumentation, Woburn, MA, USA). At 12 h post-injection, the mice were sacrificed. The tumor and normal tissues (tumor, liver, spleen, lung, kidney, and heart) were excised, followed by washing the surface with 0.9% NaCl for the *ex vivo* imaging of DiR fluorescence using a Maestro™ *in vivo* imaging system (Cambridge Research & Instrumentation, Woburn, MA, USA). The mice treated without 0.2 mL of the DiR-PLA NPs/MMC-SPC were used for comparison.

### *In vivo* anticancer effect

Kunming mice aged 4 to 5 weeks (clean class, 18 to 22 g) were supplied by Xiamen University Laboratory Animal Center and used in this study. Subcutaneous tumors were established in the mice by subcutaneous inoculation of 5 × 10^6^ H_22_ cells in the right axillary region of the mice before the treatment. H_22_ tumors were not hormone dependent and easily grown in the subcutaneous layer of mice. The tumors were allowed to grow for 1 week after tumor transplantation, after that, the treatment was performed. The H_22_ tumor-bearing mice were randomly divided into 4 groups (10 mice per group): group 1 for 0.9% NaCl, group 2 for PLA NPs/SPC, group 3 for free MMC injection, and group 4 for PLA NPs/MMC-SPC. The mice were intravenously administrated at 4 mg/kg (equivalent MMC concentration) every 2 days for 3 times. Each mouse was earmarked and followed individually throughout the whole experiments. The tumor size and body weight were then monitored every 2 days. The mice were euthanized on day 12. The greatest longitudinal diameter (length) and the greatest transverse diameter (width) of the tumor were measured using a vernier caliper, and the tumor volume was calculated using length × width^2^ × 0.5.

## Results and discussion

### Preparation and characterization of PLA NPs/MMC-SPC

**Figure 2:**
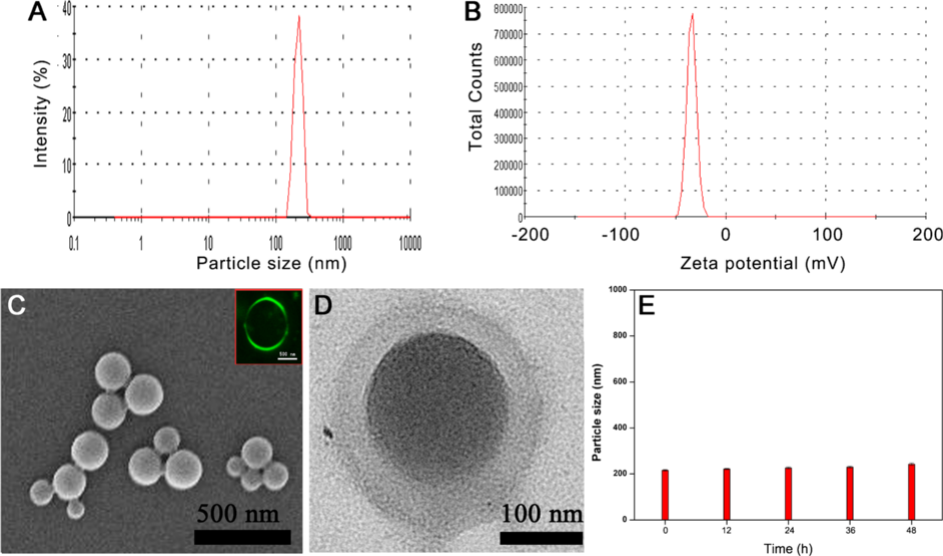
**Characteristics of the hybrid PLA NPs/MMC-SPC. (A)** Particle size with particle size distribution. **(B)** Zeta potential. **(C)** SEM image. Inset: confocal laser scanning microscopy images of the hybrid PLA NPs/FITC-MMC-SPC (FITC was conjugated with MMC via a thiourea linkage). Scale bar = 500 nm. **(D)** TEM image. **(E)***In vitro* stability in PBS at 37°C. Data are presented as mean ± SD (*n* =3).

In this study, a single-step dialysis method by addition of the amphiphilic MMC-SPC complex to the PLA polymer solution in DMF was used to form the hybrid PLA NPs/MMC-SPC. DMF is water miscible and a good solvent for the PLA polymer and MMC-SPC complex. PLA and MMC-SPC were first added to DMF (organic phase) and were then dialyzed against water (aqueous phase). After the exchange of the organic phase and the aqueous phase under continuous dialysis process, the PLA polymer and MMC-SPC complex precipitated to form the PLA NPs/MMC-SPC with a core-shell structure, and the amphiphilic MMC-SPC complex was adsorbed and self-assembled on the surface of the PLA core by hydrophobic interaction, as illustrated in Figure 1 (more details are shown in Figure 2). More importantly, the drug-phospholipid complex was not only used as a structural element of the polymer-lipid hybrid nanoscaled drug delivery systems but also served as a stabilizer/emulsifier to facilitate and stabilize their formation.

The hybrid PLA NPs/MMC-SPC comprised a hydrophobic PLA core and an amphiphilic MMC-SPC shell. Once intravenously administrated, the polymer-lipid hybrid NPs were accumulated at the tumor site via the enhanced permeability and retention (EPR) effect. And then, the polymer-lipid hybrid NPs were internalized by the cells via endocytosis. Lastly, MMC was released from the polymer-lipid hybrid NPs and delivered to the nuclei, presenting the anticancer activity.

The effect of the MMC-SPC concentration on the size, zeta potential, and encapsulation efficiency of the hybrid PLA NPs/MMC-SPC was investigated. As shown in Additional file 1: Figure S1 in supporting information, when the concentration of MMC-SPC increased, the particle size of the hybrid PLA NPs/MMC-SPC initially decreased and then increased. It was believed that at the appropriate MMC-SPC concentration (0.36 mg/mL), the amount of the amphiphilic MMC-SPC complex was in the range to able to cover the surface of the hydrophobic PLA core. However, when the MMC-SPC concentration was too high, the excess of the MMC-SPC complex might participate in the self-organization of the large size, vesicle-like structures (50 to 500 nm) [7,22]. Therefore, in the following studies, a MMC-SPC concentration of 0.36 mg/mL was used to prepare the hybrid PLA NPs/MMC-SPC.

Table 1 shows a comparison of the characteristics (particle size, particle size distribution, zeta potential, drug encapsulation efficiency, and drug loading content) of PLA NPs/MMC-SPC (MMC-SPC not only used as a drug preparation but also served as an emulsifier) and PLA NPs/MMC (PVA used as an emulsifier), from which it can be seen that as emulsifier, SPC had several aspects of advantages over the traditional PVA. Firstly, the particle size of the PLA NPs/MMC-SPC was approximately 200 nm with PDI < 0.200, while the particle size of the PLA NPs/MMC was approximately 300 nm with PDI > 0.200. It can be explained that the MMC-SPC complex could lower the surface energy of the oil-water interface and thus facilitate/stabilize the NP formulation. Secondly, the zeta potential of the PLA NPs/MMC-SPC was approximately -35 mV, while the zeta potential of the PLA NPs/MMC was found to be about -20 mV. It can be attributed to the presence of the negative charge from the phosphorous group in the SPC molecule. Lastly, to prepare the NPs with the similar drug encapsulation efficiency (to easily compare the drug loading content of the two formulations, we made the drug encapsulation efficiency for the two formulations about equal by varying the weight of the input drug, lipid, and polymer), the drug loading content of the PLA NPs/MMC-SPC was approximately sixfold higher that of the PLA NPs/MMC. MMC-SPC may serve as a function of the structural element of the nanoscaled drug delivery systems, providing a significant higher retaining of the loaded drug.

**Table 1:** Particle size, PDI, zeta potential, drug encapsulation efficiency, and drug loading content of the PLA NPs/MMC-SPC and PLA NPs/MMC

	**Particle size (nm)**	**PDI**	**Zeta potential (mV)**	**Drug encapsulation efficiency (%)**	**Drug loading content (%)**
PLA NPs/MMC-SPC	205.3 ± 5.7	0.110 ± 0.021	-36.3 ± 2.9	37.6 ± 2.9	10.3 ± 2.4
PLA NPs/MMC	327.1 ± 12.9	0.320 ± 0.047	-18.7 ± 1.5	34.5 ± 3.8	1.7 ± 0.4

Data are presented as mean ± SD (*n* =3).

As it is reported, a particle size (200 to 300 nm) may be suited for the EPR effect [39,40], providing a passive targeting. A high zeta potential (lower than -30 mV or higher than 30 mV) can provide an electrostatic repulsion to avoid the aggregation of the particles [41]. A high drug loading content is essential for intravenous administration, which will reduce the dosage of carriers and the possible side effects to the patients [42,43]. Thus, SPC had much higher emulsification efficiency than PVA. Moreover, SPC was a natural product, and the PVA was a chemical. The former can thus cause less side effects than the latter. All of the results demonstrated a prospect of the useful nanoscaled drug delivery systems (PLA NPs/MMC-SPC) with a nanoscaled particle size, a narrow particle size distribution (Figure 2A and Table 1), a high zeta potential (Figure 2B and Table 1), a spherical shape (Figure 2C,D), a well-stability (Figure 2E, discussed as below) and a high drug loading content (Table 1) for drug delivery.

### *In vitro* stability

The clinical use of the PLA NPs as intravenous drug delivery systems is limited due to their poor physiological stability. We thus investigated if the hybrid PLA NPs/MMC-SPC can overcome this drawback. As shown in Figure 2E, the hybrid PLA NPs/MMC-SPC exhibited a structural stability under physiological conditions as demonstrated by no significant particle size change over 48 h. We speculated that the presence of the phospholipid complex on the surface of the hybrid PLA NPs/MMC-SPC prevented the aggregation of the nanoscaled drug delivery systems.

### *In vitro* drug release

**Figure 3:**
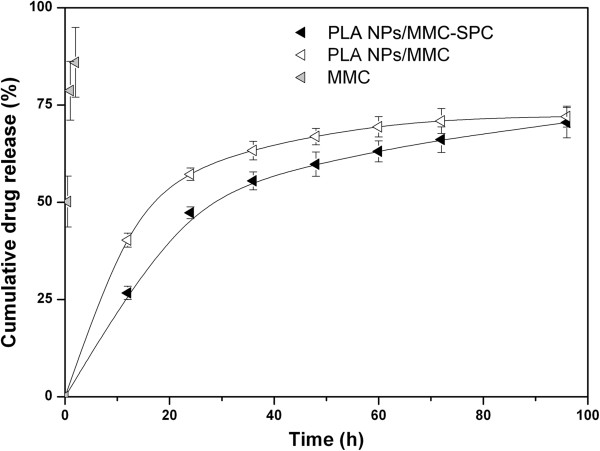
***In vitro *drug release of the hybrid PLA NPs/MMC-SPC.** Time-dependent drug release from the PLA NPs/MMC-SPC, PLA NPs/MMC, and free MMC in PBS at pH 7.4. Data are presented as mean ± SD (*n* =3).

The *in vitro* drug release profiles of the PLA NPs/MMC-SPC and PLA NPs/MMC were monitored as a function of time. As clearly shown in Figure 3, the amount of MMC released from the PLA NPs/MMC-SPC is no more than 30% over 12 h, whereas that of MMC released from the PLA NPs/MMC is approximately 50%. The result indicated that the PLA NPs/MMC-SPC were more efficient than the PLA NPs/MMC in reducing the premature burst release (minimizing the initial drug release in the blood circulation). The difference was possibly due to the fact that both the hydrophobic interaction of PLA polymer molecules and SPC lipid molecules and the electrostatic interaction of SPC lipid molecules and MMC drug molecules conferred the effective integrity of the hybrid PLA NPs/MMC-SPC. The MMC-SPC complex trapped on the surface of the hybrid NPs would suffer the dissociation, and subsequently, MMC as a free form could diffuse from the hybrid NPs. We believed that the introduction of drug-phospholipid complex which not only acted as a bridge of drug loading but also served as a control of drug release, could endow the drug not only to increase the drug loading content but also to prolong the drug release period, which might actually contribute to the improved therapeutic efficacy and the reduced side effects.

### *In vitro* cellular uptake

**Figure 4:**
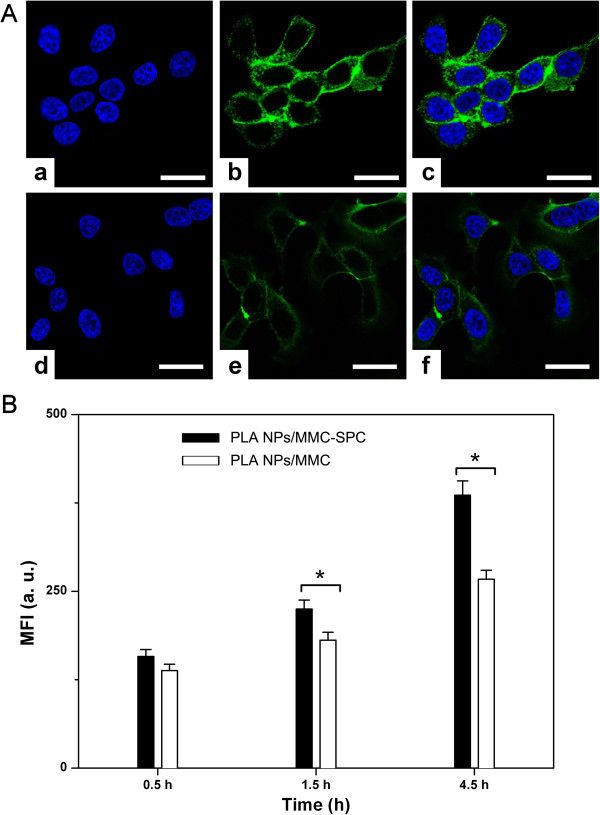
***In vitro *cellular uptake of the hybrid PLA NPs/MMC-SPC. (A)** Confocal laser scanning microscopy images of H_22_ cells incubated with the **(a, b, and c)** coumarin-6-PLA NPs/MMC-SPC and **(d, e, and f)** coumarin-6-PLA NPs/MMC at the equivalent coumarin-6 concentration for 6 h at 37°C. **(a and d)** Left column showed the blue fluorescence from the cell nuclei (the cell nuclei were stained with Hoechst 33258 (Sigma-Aldrich, St. Louis, MO, USA)). **(b and e)** Middle column showed the green fluorescence from the NPs (the NPs were labeled with coumarin-6). **(c and f)** Right column is the merged picture of the left column and right column. All the scale bars represented 25 μm. All images were taken under identical instrumental conditions. **(B)** Flow cytometer tests of H_22_ cells incubated with the coumarin-6-PLA NPs/MMC-SPC and coumarin-6-PLA NPs/MMC (equivalent coumarin-6 concentration) for 0.5, 1.5, and 4.5 h. Data are presented as mean ± SD (*n* =3). *P < 0.05 (two-tailed Student's *t*-test).

We selected H_22_ cell as the model to evaluate the cellular uptake of the coumarin-6-labeled PLA NPs/MMC-SPC and PLA NPs/MMC at the equivalent concentration of coumarin-6 using confocal laser scanning microscopy. To better compare the cell internalization among the coumarin-6-PLA NPs/MMC-SPC and coumarin-6-PLA NPs/MMC, the images were taken by harmonizing the parameters such as laser power, sensitivity, offset, and gain constant during the cell imaging procedure. As shown in Figure 4A, the green fluorescence was observed within H_22_ cells after 6 h of incubation, indicating the internalization of the PLA NPs/MMC-SPC and PLA NPs/MMC. More importantly, the significantly higher fluorescence intensity was observed in the cytoplasm of H_22_ cells treated with the coumarin-6-PLA NPs/MMC-SPC compared to those treated with the coumarin-6-PLA NPs/MMC with the same incubation time under the identical instrumental conditions. The result can be explained by the existence of SPC on the surface of the hybrid PLA NPs/MMC-SPC. Since SPC was an amphiphilic molecule composing of a hydrophilic head and a hydrophobic tail and also was similar to the phospholipids of the cell membrane in the structure component; thus, the cellular uptake of the hybrid PLA NPs/MMC-SPC was facilitated by the mutual interaction between the hybrid PLA NPs/MMC-SPC and the cell membrane.

### Flow cytometry

To more precisely understand how the composition of formulations affected the cell internalization of the NPs, H_22_ cells were incubated with the coumarin-6-PLA NPs/MMC-SPC and coumarin-6-PLA NPs/MMC and subsequently analyzed the cells by flow cytometry. As clearly shown in Figure 4B, both the coumarin-6-PLA NPs/MMC-SPC and coumarin-6-PLA NPs/MMC indeed showed a time-dependent cell uptake. The cellular uptake of the coumarin-6-PLA NPs/MMC-SPC was not significantly different from that of the coumarin-6-PLA NPs/MMC-SPC after incubation of 0.5 h. In contrast, the cellular uptake of the coumarin-6-PLA NPs/MMC-SPC was significantly higher than that of the coumarin-6-PLA NPs/MMC after incubation of 1.5 and 4.5 h. The different phenomenon could be explained by cell penetration rate of the nanoscaled drug delivery systems depending on the NP concentration differences between the internal and external environment of the cell membrane or the interaction of the NPs and the cell membrane. It should be concluded that in the case of the incubation of a short period of time, the NP concentration differences between the internal and external environment of the cell membrane played a main role in the cell penetration rate of the nanoscaled drug delivery systems; however, in the case of the incubation of a long period of time, the interaction of the NPs and the cell membrane played a vital role.

Consistent with the observations by confocal laser scanning microscopy, the result of flow cytometry confirming that enhanced cellular internalization (45.6% increased for 4.5 h) of the PLA NPs/MMC-SPC compared with the PLA NPs/MMC. The lipophilicity and liposolubility of SPC on the surface of the hybrid PLA NPs/MMC-SPC perhaps increased the endocytosis and facilitated the passive delivery of the PLA NPs/MMC-SPC NPs to the interior of the cells.

### Intracellular drug delivery

**Figure 5:**
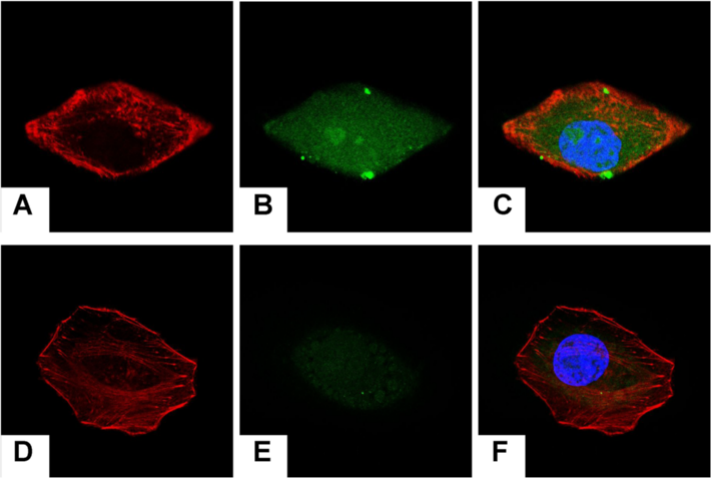
**Intracellular drug delivery of the hybrid PLA NPs/MMC-SPC.** Confocal laser scanning microscopy images of H_22_ cells incubated with the **(A,B,C)** coumarin-6-PLA NPs/FITC-MMC-SPC and **(D,E,F)** coumarin-6-PLA NPs/FITC-MMC at the equivalent coumarin-6 concentration for 12 h at 37°C. **(A,D)** Left column showed the red fluorescence from the cytoskeleton (the cell nuclei were stained with rhodamine-phalloidin). **(B,E)** Middle column showed the green fluorescence from the drugs (the drugs were labeled with FITC). The merged image of the images in left column and middle column is shown in **(E,F)** right column. The nuclei were stained with Hoechst 33258 (Sigma-Aldrich, St. Louis, MO, USA) (blue).

The fate of MMC after entering the cell is important for subsequent induction of cell death/apoptosis in the nuclei [44,45]. To investigate whether the MMC molecules loaded within the hybrid PLA NPs/MMC-SPC could be transported to the nuclei, H_22_ cells were treated with the PLA NPs/FITC-MMC-SPC, and the intracellular drug delivery is shown in Figure 5. The blue fluorescence resulting from the Hoechst 33258 (Sigma-Aldrich, St. Louis, MO, USA) staining served to label the nuclei, the red fluorescence resulted from the rhodamine-phalloidin staining served to label the cytoskeleton, and the green fluorescence represented the FITC-MMC.

For both the PLA NPs/FITC-MMC-SPC and PLA NPs/FITC-MMC, the green fluorescence was presented in the cell. This finding was similar to the reported work [46]. It was also reported that the premature released FITC or FITC-MMC could be not internalized by the cells [37]. To the result, we proposed two possible reasons, one was that the intracellular drug release and transport played a dominate role. A part of drug was released from the NPs and transported inside the cells. Alternatively, the other one was that the intracellular drug accumulation and diffusion played a main role. A high nuclear accumulation of FITC-labeled MMC resulted in the subsequent diffusion to other cytoplasmic areas. Regardless of either mechanism, the result confirmed that the drug delivered to the nuclei resulted from the effective cellular uptake, followed by the efficient intracellular internalization and accumulation of drug.

It should be noted that, the fluorescence signals of H_22_ cells incubated with the PLA NPs/FITC-MMC-SPC were significantly increased compared with those incubated with the PLA NPs/FITC-MMC at the same incubation time under the identical instrumental conditions, the result combined with the *in vitro* cellular uptake further confirmed that the interaction of SPC on the surface of the hybrid PLA NPs/FITC-MMC-SPC, and the cell membrane enhanced the cellular uptake of the nanoscaled drug delivery systems, hence, increased the intracellular internalization and accumulation of FITC-labeled MMC. A sufficient intracellular drug concentration was essential for an enhanced anticancer activity. Escape of the delivered MMC from endo/lysosome into nuclei was important as the target site of MMC was nuclear DNA and MMC could interrupt its function. Although more studies were needed to better understand the nuclear radiolabeled MMC delivery and the underlying mechanism, the result did suggest that our NPs were capable of effectively mediating intracellular drug delivery.

### *In vitro* cell viability

**Figure 6:**
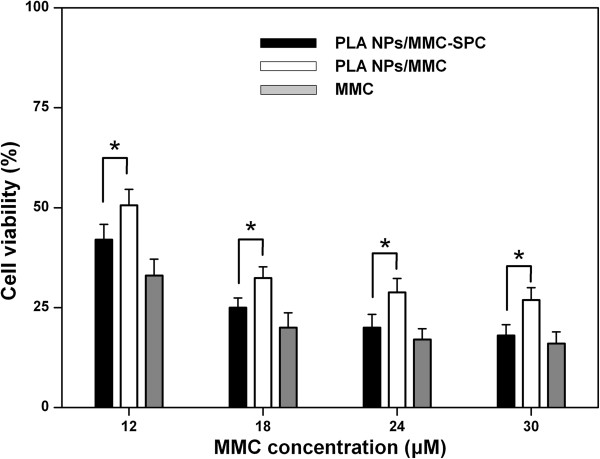
***In vitro *cell viability of the hybrid PLA NPs/MMC-SPC.** Concentration-dependent cell viability of H_22_ cells treated with the PLA NPs/MMC-SPC, PLA NPs/MMC, and free MMC after incubation of 24 h. Data are presented as mean ± SD (*n* = 6). *P < 0.05 (two-tailed Student's *t*-test).

The efficacy of the PLA NPs/MMC-SPC to defeat cancer cells is reflected by their cytotoxicity. As shown in Figure 6, the PLA NPs/MMC-SPC and PLA NPs/MMC showed the concentration-dependent cytotoxicity against H_22_ cells for 24 h. It was straightforward to understand that the lower cell viability corresponds to the higher concentration of drugs. Notably, the PLA NPs/MMC-SPC significantly reduced the cell viability compared to the PLA NPs/MMC at the same drug concentration. As it is reported, the drug-loaded polymer NPs might be internalized by endocytosis due to their nanoscaled particle size [47]. Nevertheless, owing to the affinity of SPC to cells, the polymer-lipid hybrid NPs might further enhance the internalization of drug. Therefore, the result can be ascribed to the enhanced cellular uptake of the nanoscaled drug delivery systems (see Figure 4) and the increased intracellular accumulation of the drugs (see Figure 5), which result in the improved anticancer activity.

Besides, the cytotoxicity PLA NPs/MMC-SPC were not as effective as the free MMC against H_22_ cells at an equivalent MMC concentration (Figure 6), which was most probably because of the prolonged drug release from the hybrid drug-loaded NPs (see Figure 3). Such a sustained and prolonged drug release of the PLA NPs/MMC-SPC might result in the progressive increase of intracellular drug concentration for cell death. On the contrary, the free drugs can be rapidly transported into cells by passive diffuse owing to the driving force of a pH and concentration gradient and directly inhibit the cell growth without the drug release [46]. Although the PLA NPs/MMC-SPC induced a decline in the cytotoxicity of MMC, the introduction of the nanoscaled drug delivery systems could increase the passive targeting efficiency *in vivo* (discussed below). Once accumulated at the tumor site, the polymer-lipid hybrid NPs as a whole would easily into the interior of the tumor cells to exert the pharmacological effects.

### *In vivo* stability

**Figure 7:**
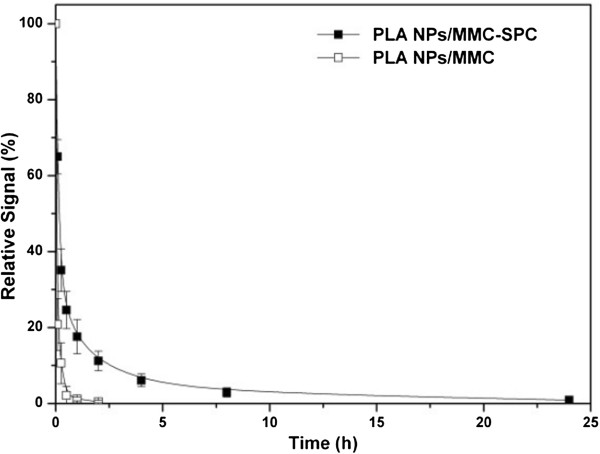
***In vivo *blood clearance of the hybrid PLA NPs/MMC-SPC.** Rats were intravenously injected with the DiR-PLA NPs/MMC-SPC and DiR-PLA NPs/MMC. Data are presented as mean ± SD (*n* = 6).

The *in vivo* stability of PLA NPs/MMC-SPC was evaluated by monitoring the blood clearance of the PLA NPs/MMC-SPC (Figure 7). A hydrophobic near-infrared fluorescent dye DiR was loaded within the PLA NPs/MMC-SPC as a probe since the excitation and emission wavelengths of DiR do not overlap with the autofluorescence of blood, allowing its concentration to be measured directly from the whole blood fluorescence [38]. The intravenously injected DiR-PLA NPs/MMC was rapidly cleared from the systemic circulation; the fluorescence signal of the DiR-PLA NPs/MMC in the blood was hardly detectable in as little as 0.5 h after the intravenous injection, which was expected based on their rapid aggregations in plasma. In contrast, the DiR-PLA NPs/MMC-SPC had a slower clearance profile, indicating that MMC-SPC reduced the nonspecific interactions with the plasma protein to some extent. Although more studies are needed to better understand the pharmacokinetics of MMC, the result did suggest that the PLA NPs/MMC-SPC were capable of effectively enhancing the blood retention in the systemic circulation.

### *Ex vivo* fluorescence imaging

**Figure 8:**
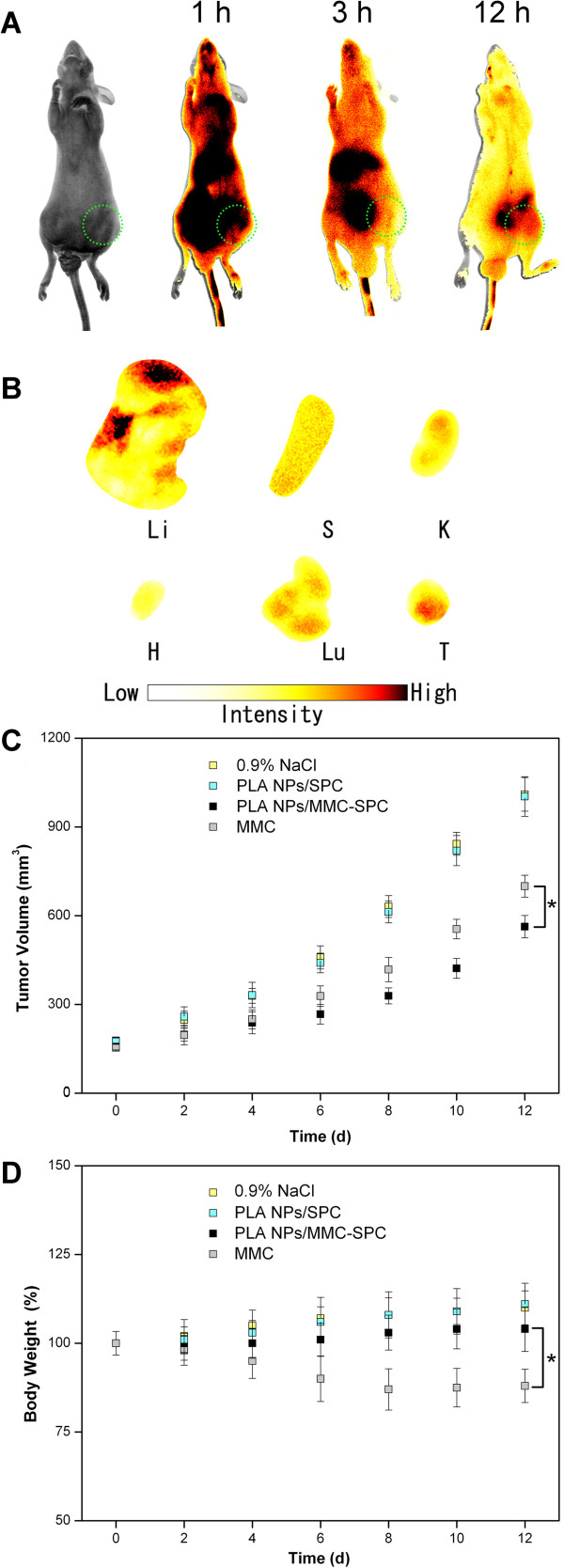
***In vivo *fluorescence imaging and *in vivo *anticancer effect of the hybrid PLA NPs/MMC-SPC. (A)***In vivo* fluorescence imaging of H_22_ tumor-bearing mice after intravenous injection of the DiR-PLA NPs/MMC-SPC at 1, 3, and 12 h post-injection. **(B)***Ex vivo* fluorescence imaging of H_22_ tumor-bearing mice after intravenous injection of the DiR-PLA NPs/MMC-SPC at 12 h post-injection (Li, liver; S, spleen; K, kidney; H, heart; Lu, lung; T, tumor). **(C)** The change of tumor volumes of H_22_ tumor-bearing mice treated with PBS, PLA NPs/SPC, PLA NPs/MMC-SPC, and MMC. **(D)** The change of body weights of H_22_ tumor-bearing mice treated with 0.9% NaCl, PLA NPs/SPC, PLA NPs/MMC-SPC, and MMC.

To reveal the potential of such hybrid NPs as a MMC delivery system *in vivo*, near-infrared fluorescent probe DiR was incorporated into the PLA NPs/MMC-SPC. Mice bearing murine hepatoma H_22_ tumor were intravenously administrated with DiR-PLA NPs/MMC-SPC via the lateral tail vein. The *ex vivo* biodistribution of the DiR-PLA NPs/MMC-SPC micelles was then investigated at 12 h post-injection. Organs from a control mouse without injection of the DiR-PLA NPs/MMC-SPC were collected and used as controls to subtract the autofluorescence background in various tissues. Figure 8A shows the real-time images of the DiR-PLA NPs/MMC-SPC in the H_22_ tumor-bearing nude mice. *In vivo* imaging showed that the DiR-PLA NPs/MMC-SPC were clearly observed in the body, and then, the fluorescent signals gradually became weaker as the time elapsed. At 12 h post-injection, the high-intensity fluorescent signals of the DiR-PLA NPs/MMC-SPC were observed at the tumor site. As shown in Figure 8B, a relatively strong fluorescence in the liver was observed compared to other organs, which was likely attributed to the nonspecific clearance of the NPs by the reticuloendothelial system (RES). The result was in agreement with the report in the literature [48]. In addition, the strong fluorescence was observed at the tumor site, demonstrating some accumulation of the DiR-PLA NPs/MMC-SPC in the tumor tissue. The result could be explained by the particle size-mediated EPR effect [4,49]. However, on the basis of the EPR effect, the targeting efficiency of the PLA NPs/MMC-SPC might be further increased by the functionalization of a long-circulation agent (such as PEG) coordinated with a targeting ligand (such as folate or cRGD). The relevant studies were still under investigation.

### *In vivo* anticancer effect

Next, we examined the anticancer effect of the PLA NPs/MMC-SPC after the accumulation at the tumor site. As shown in Figure 8C, the intravenous injection of the PLA NPs/MMC-SPC and free MMC inhibited the tumor growth, whereas the MMC-free PLA NPs/SPC did not slow the tumor growth. The result showed that MMC, the delivery of which was mediated by the hybrid PLA NPs/MMC-SPC, was responsible for the tumor growth inhibition. More importantly, the tumor growth of mice treated with the PLA NPs/MMC-SPC was much slower than that of mice treated with the free MMC, and the difference became more significant after day 12. In addition, the loss of body weight in mice accompanied the treatment with the free MMC in this study (Figure 8D) but was not found in the treatment with the hybrid PLA NPs/MMC-SPC. All of the results suggested that compared with the free MMC, the hybrid PLA NPs/MMC-SPC showed a significantly enhanced therapeutic efficacy while reducing the side effect of chemotherapy drug.

We concluded that the following reasons might be involved. Firstly, the free MMC is cleared too rapidly, and thus, the low concentrations of drugs in the tumor tissues will result in suboptimal therapeutic effects [2]. On the contrary, the EPR effect and the sustained release of the nanoscaled drug delivery systems may result in a sufficient intracellular drug. Secondly, the nanoscaled drug delivery systems can help the loaded drug effectively enters the interior of cells by endocytosis. Lastly, the lipophilicity and liposolubility of SPC on the surface of the polymer-lipid hybrid NPs efficiently help the NPs transport from the surrounding water-soluble environment to the lipid-rich cell membrane and enter the internal environment of the cells, leading to the increased internalization and accumulation of drug inside the cells. Therefore, the present study suggested that after the intravenous administration, the hybrid PLA NPs/MMC-SPC were useful in significantly improving the anticancer effect of MMC while reducing its toxicity compared to the MMC injection for clinical treatment.

## Conclusions

We have developed the MMC-loaded polymer-lipid hybrid NPs for sustained and controlled release of MMC by a single-step self-assembly. The composition of the PLA NPs/MMC-SPC not only affected their drug release but also influenced their cellular uptake and anticancer efficacy. We concluded that natural SPC had great advantages over traditional PVA with less burst drug release, more cellular uptake, and higher anticancer efficacy. The PLA NPs/MMC-SPC might efficiently ensure the nuclear delivery of MMC to induce cell death. More importantly, the PLA NPs/MMC-SPC showed the improved therapeutic efficiency compared with the free MMC injection. All of the results suggested that the MMC-loaded polymer-lipid hybrid NPs have promising potential as attractive and practical nanoscaled MMC delivery systems for cancer therapy.

## Competing interests

The authors declare that they have no competing interests.

## Authors’ contributions

YY and YL conceived and carried out the experiments, analyzed the data and wrote the paper. ZH designed the study, supervised the project, analyzed the data and wrote the paper. MJ, XY, HW, and YH assisted in the synthesis and characterizations of the NPs. HW, JL, and SW assisted in the biological evaluations of the NPs. YL, ZH, and LX provided the insightful comments regarding the mechanism of drug delivery and cancer therapy. All authors read and approved the final manuscript.

## Authors’ information

Yunfeng Yi and Yang Li are co-first authors.

## Supplementary Material

Additional file 1: Figure S1 Effect of the MMC-SPC concentration on the particle size, zeta potential, and drug encapsulation efficacy of the hybrid PLA NPs/MMC-SPC. Data are presented as mean ± SD (*n* =3). Click here for file
