# Supplementary material for: Single-step assembly of polymer-lipid hybrid nanoparticles for mitomycin C delivery
Source: Nanoscale Res Lett. 2014 Oct 8;9(1):560. doi: 10.1186/1556-276X-9-560 (PMC4198073; doi:10.1186/1556-276X-9-560)
Supplement: Additional file 1: Figure S1 — Effect of the MMC-SPC concentration on the particle size, zeta potential, and drug encapsulation efficacy of the hybrid PLA NPs/MMC-SPC. Data are presented as mean ± SD (n =3). [file 1556-276X-9-560-S1.doc]

***Additional file***


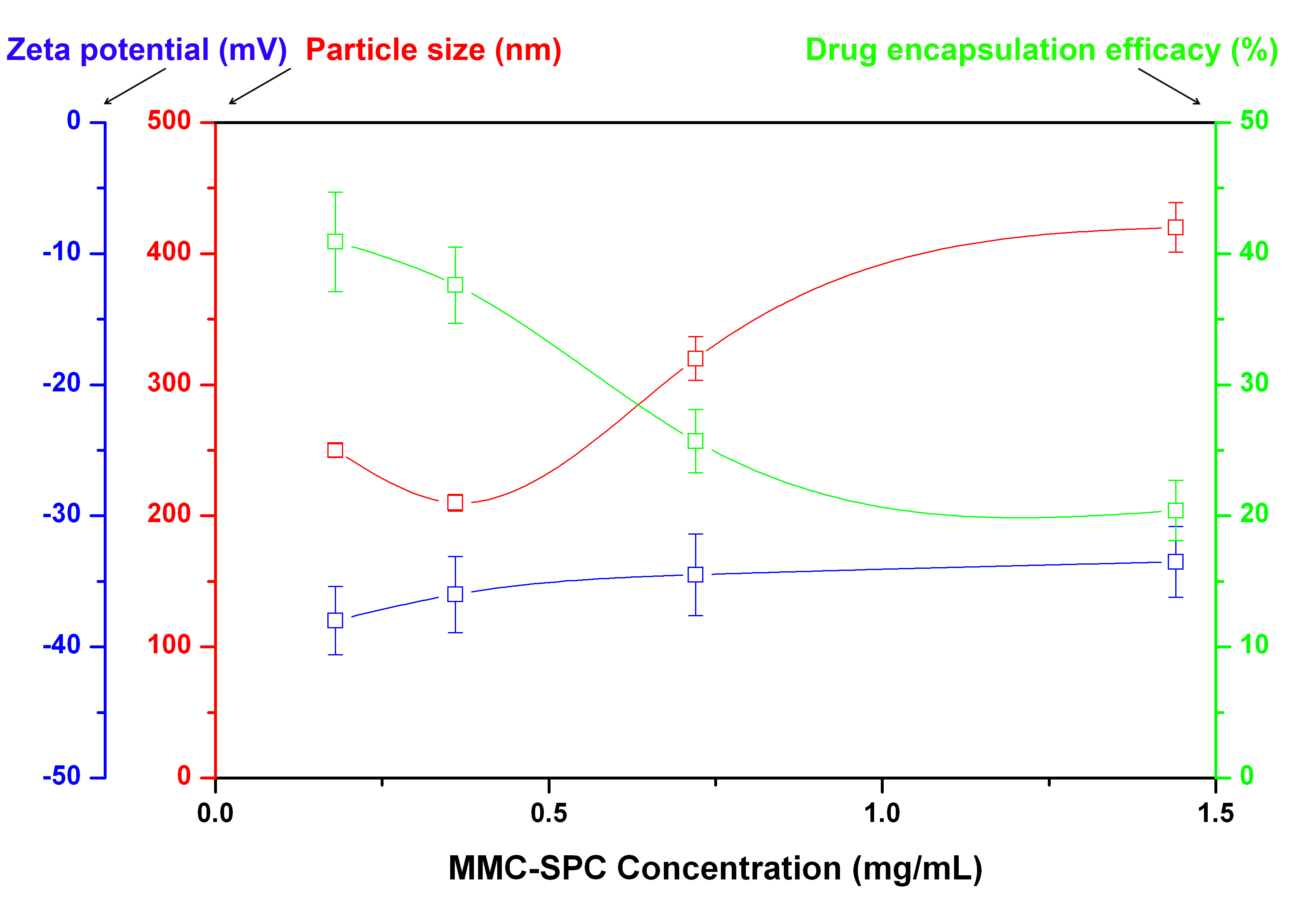


Figure S1. Effect of the MMC-SPC concentration on the particle size, zeta potential and drug encapsulation efficacy of the hybrid PLA NPs/MMC-SPC. Data are presented as mean ± SD (n =3).
